# Gender Differences in Sexual Coercion Perpetration: Investigating the Role of Alcohol-use and Cognitive Risk Factors

**DOI:** 10.1177/08862605211006360

**Published:** 2021-04-14

**Authors:** Véronique Bonneville, Dominique Trottier

**Affiliations:** 1 Université du Québec en Outaouais, Gatineau, Québec, Canada; 2 Philippe-Pinel National Institute of Forensic Psychiatry, Montreal, Canada

**Keywords:** sexual coercion perpetration, alcohol-use, alcohol-related risk factors, cognitive risk factors, gender differences

## Abstract

Studies have shown that alcohol is involved in 50 to 75% of all sexual coercion situations. Significant associations have been established between alcohol-use and sexual coercion perpetration and cognitive factors have been proposed to play an important role in this association. However, the current knowledge on the relationship between alcohol-use, cognitive factors, and sexual coercion perpetration is mostly based on male samples. Therefore, the purpose of this article is to investigate gender differences associated with the role of alcohol-use and cognitive factors in sexual coercion perpetration. To do so, 742 participants (562 women, 180 men) completed an online questionnaire assessing (1) alcohol-use, (2) perpetration of sexual coercion, and (3) cognitions related to sexuality or alcohol (misperception of sexual intent, alcohol-related expectancies, alcohol-related rape myth acceptance [RMA]). Results revealed that (1) for both men and women, alcohol-use as well as cognitive variables allowed to discriminate perpetrators from non-perpetrators, (2) perpetrators, whether male or female, did not differ significantly on any of the risk factors, except for alcohol-related RMA, (3) a prediction model that considered cognitive variables, as well as alcohol-use significantly contributed to the explanation of both male and female sexual coercion, and (4) the prediction model explained three times the amount of variance in sexual coercion perpetrated by men compared to women. On the one hand, these results highlight similarities in risk factors towards sexual coercion perpetration for both men and women. Perpetrators, regardless of their gender, seem to exhibit similar alcohol-use, alcohol-related expectancies, and tendencies to misinterpret sexual intent, making these risk factors potential prevention and intervention targets for both genders. On the other hand, these results emphasize the need to break away from male-based sexual coercion explanatory models and consider other variables towards a better understanding of female sexual coercion perpetration.

## Introduction

Sexual coercion refers to the use of explicit (e.g., physical force, weapons, threats) or implicit (e.g., lies, manipulation) constraints in order to obtain any type of sexual contact from an unwilling individual (Schatzel-Murphy et al., 2009). Numerous studies have established associations between distal and proximal measures of alcohol consumption and sexual coercion (See Abbey et al., 2014 for a review). More specifically, studies have shown that alcohol is involved in 50 to 75% of all sexual coercion situations (Abbey et al., 2004; Krahé & Berger, 2013; Mohler-Kuo, et al., 2004) and that perpetrators of sexual coercion are more likely to make problematic use of alcohol than those with no history of sexual coercion (Abbey & Jacques-Tiura, 2011). However, studies linking alcohol consumption and sexual coercion generally suggest that alcohol-use needs to be considered in combination with other risk factors for sexual coercion. Among these risk factors, cognitive factors, such as misperception of sexual intent and attitudes supportive of sexual coercion have been proposed to play an important role in sexual coercion perpetration (Abbey, 2011; Abbey et al., 2004; Benbouriche, 2016). While multiple studies have linked alcohol-use, cognitive factors, and sexual coercion, most studies have focused solely on male samples. Therefore, the goal of the current study is to investigate gender differences associated with the role of alcohol-use and cognitive factors in sexual coercion perpetration.

### Cognitive Factors Associated with Sexual Coercion

#### Misperception of Sexual Intent

Interactions that lead to sex generally begin with the exchange of verbal and non-verbal cues indicating a mutual interest, which have to be perceived and interpreted by both involved parties. Studies have shown that men are more likely to perceive another’s behavioral intentions incorrectly, and tend to perceive women as being more sexually receptive than they are (Abbey, 1991; Abbey & Harnish, 1995; Farris et al., 2008). Past studies have revealed that misperception of sexual intent is associated with an increased likelihood of sexual coercion in male perpetrators (Abbey et al., 1998; Abbey et al., 2001; Farris et al., 2008). By misinterpreting a person’s actions as indicating sexual interest, one may believe that their use of sexually coercive strategies is more acceptable, even if a lack of interest is expressed later on (Benbouriche, 2016). This kind of reasoning is reinforced by widespread rape myths (“When girls are raped, it’s often because the way they said ‘no’ was unclear”) and endorsement of token resistance (women initially say “no” to sex even if they want to, to fulfill a role of sexual passivity; Burt, 1980; Canan et al., 2016; McMahon & Farmer, 2011; Muehlenhard & Rodgers, 1998). The role of misperception of sexual intent in female sexual coercion perpetration has not yet been investigated to our knowledge.

#### Rape Myth Acceptance

Rape myths can be defined as prejudicial and false beliefs regarding rape, rape victims and rape perpetrators that serve to blame the victim, remove guilt from the perpetrator, or minimize the violence that is involved in the act of raping (Bohner et al., 2006; Burt, 1980). Multiple studies have demonstrated the association between rape myth acceptance (RMA) and sexual coercion (see Trottier et al., 2019 for a meta-analysis). Some rape myths are specifically related to alcohol consumption. Thus, during a sexual interaction, certain rape myths may be activated to minimize the perpetrator’s responsibility (“It shouldn’t be considered rape if a guy is drunk and didn’t realize what he was doing;” McMahon & Farmer, 2011) or to minimize the gravity of the situation (“If both people are drunk, it can’t be rape;” McMahon & Farmer, 2011). Certain rape myths can also be used to redirect blame following a sexually coercive act (e.g., “If a girl is raped while she is drunk, she is at least somewhat responsible for letting things get out of control;” McMahon & Farmer, 2011). By redirecting blame from the perpetrator to the victim, the risk of sexual coercion increases (Abbey, 1991). Although RMA has been extensively studied in the context of male sexual coercion, a recent systematic review has highlighted the absence of studies comparing RMA between female perpetrators and non-perpetrators (Trottier, Benbouriche, Bonneville, et al., 2020).

#### Alcohol-related Expectancies

Nowadays, alcohol is a common and normalized part of social interactions and it is often associated with sexuality and aggressivity. Some of the alcohol-related expectancies to which men may adhere are that alcohol can make them more powerful, more sexual, and more aggressive (Abbey et al., 1998; Brown et al., 1980). Expectancies guide our behaviors, our interpretations, and our intentions. Studies have shown that if a man is made to believe he drank (by way of a placebo condition), he will often report effects that are associated with these expectancies (Bègue et al., 2009; Norris et al., 2002). Alcohol-related expectancies are directly associated with the probability of using sexual coercion (Norris et al., 2002). Although not much is known about the association between alcohol-related expectancies and female sexual coercion perpetration, one study focusing on adolescents has identified negative alcohol expectancies as being a predictor of sexual coercion in both male and female perpetrators (Fernandez-Fuertes et al., 2018).

### Aims and Objective

Studies examining alcohol’s role in sexual coercion have determined that alcohol-use effects are often indirect, insofar as a variety of cognitive factors should also be considered. Namely, misperception of sexual intent, alcohol-related expectancies and attitudes supportive of sexual coercion appear to be promising contributive variables. However, the majority of the available knowledge linking alcohol consumption, cognitive factors, and sexual coercion results from studies on male samples. In recent years, there has been growing interest in identifying risk factors for female sexual coercion perpetration. Some studies have suggested differences in predictors of sexual coercion between male and female perpetrators (Schatzel-Murphy et al., 2009). Others suggest that similar factors may exist, although they might work differently according to the gender (Bouffard et al., 2016). The purpose of this article is to investigate gender differences associated with the role of alcohol-use and cognitive factors in sexual coercion perpetration. Specifically, we aim to: (1) compare male non-perpetrators, male perpetrators, female non-perpetrators, and female perpetrators based on alcohol-use and cognitive variables associated with sexual coercion; and (2) determine how these variables differentially contribute to the prediction of sexual coercion in male and female perpetrators.

### Method

#### Participants

A total of 757 participants completed an online questionnaire on sexuality. Ten participants identified neither as men, nor women, and were therefore removed from the sample since they could not be included in the gender-based comparison groups (insufficient sample size)^1^. Five participants were removed for inconsistent responses. The final sample consisted of 742 French-speaking participants aged 16 to 83 (*M* = 23.49; *SD* = 7.97). Most participants were females (74.7%), Caucasian (91.2%), heterosexual (88.0%) and students (83.3%). The majority were in committed relationships (62.7%). Sample size provided adequate power to detect statistical differences (Cohen, 1988; 1992).^2^ See Table 1 for the complete sociodemographic characteristics of the total sample and subgroups.

**Table 1.** Sociodemographic Characteristics of the Total Sample and Comparison Groups.

	Total(*n* = 742)	Male non-perpetrators(*n* = 113)	Male perpetrators(*n* = 67)	Female non-perpetrators(*n* = 434)	Female perpetrators(*n* = 128)
Ethnicity (*n* (%))					
Caucasian	677(91.2%)	100(88.5%)	59(88.1%)	399(91.9%)	119(93.0%)
Afro-American/African	11(1.5%)	3(2.7%)	1(1.5%)	7(1.6%)	–
Latin-American/Hispanic	12(1.6%)	1(.9%)	4(6.0%)	5(1.2%)	2(1.6%)
Asian	17(2.3%)	3(2.7%)	1(1.5%)	12(2.8%)	1(.8%)
Indigenous/First Nation	8(1.1%)	2(1.8%)	1(1.5%)	4(.9%)	1(.8%)
Arabic	3(.4%)	1(.9%)	–	1(.2%)	1(.8%)
Sexual Orientation (*n* (%))					
Predominantly or exclusively heterosexual	653(88.0%)	97(85.8%)	58(86.6%)	391(90.1%)	107(83.6%)
Bisexual	48(6.5%)	4(3.5%)	2(3.0%)	29(6.7%)	13(10.2%)
Predominantly or exclusively homosexual	31(4.2%)	11(9.7%)	6(9.0%)	10(2.3%)	4(3.1%)
Relationship status (*n* (%))					
Committed relationship	465(62.7%)	55(48.7%)	41(61.2%)	293(67.5%)	76(59.4%)
Seeing someone	58(7.8%)	9(8.0%)	5(7.5%)	30(6.9%)	14(10.9%)
Single	213(28.7%)	49(43.4%)	21(31.3%)	106(24.4%)	37(28.9%)
Occupation (*n* (%))					
Student	618(83.3%)	95(84.1%)	49(73.2%)	361(83.2%)	113(88.3%)
Salaried employee/self-employed	110(14.6%)	14(12.4%)	17(25.4%)	64(14.7%)	14(10.9%)
Other	2(.3%)	–	–	2(.5%)	–

#### Procedure

Data for this study were collected from September 2017 to December 2018 with an online questionnaire. Participants were recruited via the SONA recruitment platform, social media (Facebook, Twitter), postings displayed in public establishments (e.g., University, CÉGEPs, municipal libraries, professional training centers), and e-mails sent to students of post-secondary education establishments and members of a provincial research association. Participants had to be at least 16 years of age to participate. No other inclusion or exclusion criteria were applied. All recruitment methods invited individuals to participate in a study on sexuality and provided a link to the study consent form and questionnaire via Limesurvey. Average completion time was 30 minutes. Participants were free to exit the questionnaire at any moment. A question was included to ensure participants were responding attentively, as well as three duplicate questions for consistent responding. After completion, participants could enter in a draw to win one of four pre-paid 50$ credit cards.

### Measures

The online questionnaire was used in the context of a larger research project. Only variables relevant to this specific study will be detailed below.

#### Alcohol-use

Three separate alcohol-use constructs were measured, using standard questions frequently encountered in the scientific literature. First, alcohol-use frequency was measured by the following question: Thinking back on your drinking habits over the past 12 months, how many day(s) per week do you drink alcohol? This question was answered on a 5-point frequency scale (*0 days*; *1 day*; *2 to 3 days; 4 to 5 days*; *6 to 7 days*), with total scores ranging from 0 to 4. A higher total score indicated more frequent alcohol consumption. Second, alcohol-use quantity was measured by the following question: “on the days when you drink, how many drinks do you usually have?” This question was answered on a 6-point scale (*I never drink*; *1 drink*; *2 drinks*; *3 drinks*; *4 drinks*; *5 drinks or more*) with total scores ranging from 0 to 5. A higher score indicated alcohol was consumed in greater quantities. Third, a question measured the frequency of alcohol consumption before sexual intercourse and was answered on a single item 5-point scale that ranged from “Never” to “Almost always or always” with total scores ranging from 1 to 5. A higher score indicated that the participant consumed alcohol more frequently before having sex.

#### Sexual Coercion

Sexual coercion history was measured using a modified French version of the *Tactics First Sexual Experiences Survey–Perpetration Form* (SES-P; Abbey et al., 2005; Benbouriche, 2016). It contained 14 items answered on a 4-point frequency scale (*Never*; *one time*; *two times*; *three times or more*) which included the use of 7 forms of sexual coercion (arguments/verbal pressure; lies/false promises; guilt/anger; giving alcohol; giving drugs; taking advantage of a person’s intoxication; physical force) to obtain two forms of sexual contact: (1) fondling, kissing, sexual touching of breasts, buttocks or intimate parts, or (2) masturbation, oral sex, vaginal or anal penetration. For this study, a dichotomous variable was created where 1 indicated that the participant had engaged in at least one sexually coercive act and 0 indicated that the participant had no history of sexual coercion. The scale showed acceptable internal consistency reliability in our sample (*α* = .78).

#### Cognitive Factors

##### Misperception of sexual intent.

Misperception of sexual intent was measured using an item from Abbey (1987): “Have you ever interpreted someone’s words or actions as indicating sexual intent when they did not?” It was answered on a dichotomous scale (*yes*; *no*).

##### Alcohol-related RMA.

A validated French version of McMahon & Farmer’s (2011) *Updated Illinois Rape Myth Acceptance Scale* (FR-IRMA; Trottier, Benbouriche, LeBlanc, et al., 2020) was used to measure participants’ RMA. The FR-IRMA is a 20-item scale, that contains 5 subscales: “She asked for it;” “It wasn’t really rape;” “He didn’t mean to;” “She lied;” “He was drunk.” Items were answered on a 5-point Likert scale ranging from 1 (*strongly agree*) to 5 (*strongly disagree*). For this study, only the subscale “He was drunk” was used to focus on alcohol-related rape myths. The subscale’s items were the following: “It shouldn’t be considered rape if a guy is drunk and didn’t realize what he was doing;” “If both people are drunk, it can’t be rape.” Scores on this subscale could range from 2–10, with higher scores indicating greater alcohol-related RMA. The subscale showed acceptable internal consistency reliability in our sample (*α* = .73).

##### Alcohol-related expectancies regarding sexuality.

Two items from Abbey et al. (1998) and adapted from Brown et al. (1980) were used to measure participants’ level of agreement with two statements (one for each gender) concerning alcohol’s effects on sexual receptivity (“Men/Women become more sexually receptive when they drink”). These items were answered on a 5-point Likert scale ranging from 1 (*strongly disagree*) to 5 (*strongly agree*).

#### Data Analytic Plan

First, analyses of variance and chi-square analyses were conducted to compare male perpetrators, male non-perpetrators, female perpetrators, and female non-perpetrators on alcohol-use and cognitive variables. Second, for both men and women, logistic hierarchical regression analyses were conducted to determine the main effects of alcohol-use and cognitive variables on sexual coercion perpetration. Following best practice (Field, 2013), two separate models were tested and compared for each gender. In the first step, alcohol-use variables were entered, and in the second step, alcohol-use variables were entered along with cognitive risk factors.

### Results

#### Preliminary Analyses

Preliminary analyses allowed for assumption violation testing. For analyses of variance, the normality (skewness) and homogeneity of variance assumptions were violated. Validated corrections were applied (Pek et al., 2018). Specifically, normality was addressed using bootstrapping for post-hoc testing, while Welch’s *F* was used to correct homogeneity. For logistic regression analyses, all assumptions were met.

#### Sexual Coercion Prevalence

In our sample, 26.9% of participants reported at least one instance of sexual coercion perpetration. A significantly higher proportion of men (37.9%) reported a history of sexual coercion perpetration compared to women (22.8%; *x*^2^(1) = 16.18, *p* < .001).

#### Group Comparisons

Analyses of variance were conducted to compare: (1) male perpetrators; (2) male non-perpetrators; (3) female perpetrators; (4) female non-perpetrators on alcohol-use frequency and quantity, alcohol-use before sex, alcohol-related expectancies regarding male and female sexuality, alcohol-related RMA and misperception of sexual intent. Results showed significant between-group differences on all variables except for alcohol-related expectancies regarding male sexuality (see Table 2 for complete results). Tukey post-hoc tests were conducted to further examine between-group significant differences.

**Table 2.** Group Comparisons for All Study Variables.

	Male Non-perpetrators (*N* = 113)	Male Perpetrators(*N* = 67)	Female Non-perpetrators(*N* = 434)	Female Perpetrators(*N* = 128)	Group Comparisons
	*M*(*SD*)	*M*(*SD*)	*M*(*SD*)	*M*(*SD*)	Welch’s *F*
Alcohol consumption factors					
Alcohol-use frequency	.96a (.96)	1.38b (1.11)	.90a (.89)	1.09b (.85)	5.00**
Alcohol-use before sex	1.51a (.63)	1.90b (.96)	1.66c (.71)	1.99b (.88)	9.37***
Alcohol-use quantity	2.39a,c (1.75)	2.80a (1.61)	2.04b (1.49)	2.56c (1.49)	7.52***
Cognitive factors					
Alcohol-related expectancy: Women more sexually receptive	3.22a (1.06)	3.70b,d (1.10)	3.47b,c (1.16)	3.76 d (1.14)	5.57**
Alcohol-related expectancy: Men more sexually receptive	3.33 (1.05)	3.55 (1.16)	3.49 (1.20)	3.73 (1.17)	2.55
Alcohol-related RMA	2.86a,c (1.66)	3.22a (1.79)	2.41b (.95)	2.67c (1.27)	7.41***
					*x*2
Misperception of sexual intent (%)	30.1a	65.7b	38.5a	59.4b	39.28***

*Note*. Each superscript letter denotes a subset of group categories whose proportions or means do not significantly differ from each other at the .05 level according to bootstrapped confidence intervals.

Misperception of sexual intent scores could range from 0–1, alcohol frequency scores could range from 0–4, alcohol quantity scores could range from 0–5, drinking before sex scores could range from 1–5, expectancy scores could range from 1–5, and alcohol-related RMA scores could range from 2–10.

**p < .01, ***p < .001.

**Table 3.** Predictors of Sexual Coercion in Male Perpetrators.

	*b*	*OR*	95% CI
Step 1			
Alcohol-use before sex	.48*	1.62	[1.00, 2.64]
Alcohol-use frequency	.18	1.20	[.84, 1.72]
Alcohol-use quantity	.08	1.08	[.89, 1.31]
Constant	−1.71***	.18	
*R*^2^ = .07 (Cox & Snell) .09 (Nagelkerke). Model *x*^2^(3, 181) = 12.88, *p* < .01			
Step 2			
Alcohol-use before sex	.44	1.55	[.89, 2.97]
Alcohol-use frequency	.29	1.33	[.89, 1.99]
Alcohol-use quantity	–.03	.97	[.78, 1.21]
Misperception of sexual intent	1.47***	4.35	[2.18, 8.69]
Alcohol-related expectancy regarding female sexuality	.77**	2.17	[1.22, 3.87]
Alcohol-related expectancy regarding male sexuality	–.50	.61	[.34, 1.09]
Alcohol-related RMA	.16	1.17	[.96, 1.44]
Constant	−3.62***	−3.23	
*R*^2^ = .21 (Cox & Snell) .29 (Nagelkerke). Block *x*^2^(4, 181) = 30.70, *p* < .001			

*Note*. *OR* = Odds ratio; CI = Confidence interval.

*p < .05, **p < .01, ***p < .001.

**Table 4.** Predictors of Sexual Coercion in Female Perpetrators.

	*b*	*OR*	95% CI
Step 1			
Alcohol-use before sex	.43**	1.54	[1.15, 2.06]
Alcohol-use frequency	–.01	.99	[.77, 1.28]
Alcohol-use quantity	.15*	1.17	[1.01, 1.35]
Constant	−2.35***	.10	
*R*^2^ = .04 (Cox & Snell) .06 (Nagelkerke). Model *x*^2^(3, 556) = 21.64, *p* < .001			
Step 2			
Alcohol-use before sex	.37*	1.45	[1.08, 1.96]
Alcohol-use frequency	.03	1.03	[.79, 1.35]
Alcohol-use quantity	.15	1.16	[1.00, 1.34]
Misperception of sexual intent	.71**	2.02	[1.33, 3.08]
Alcohol-related expectancy regarding female sexuality	.18	1.20	[.87, 1.66]
Alcohol-related expectancy regarding male sexuality	–.07	.93	[.68, 1.27]
Alcohol-related RMA	.21*	1.23	[1.03, 1.48]
Constant	−3.55***	.03	
*R*^2^ = .07 (Cox & Snell) .11 (Nagelkerke). Block *x*^2^(4, 556) = 20.61, *p* < .001			

*Note*. *OR* = Odds ratio; CI = Confidence interval.

*p < .05, **p < .01, ***p < .001.

When comparing female non-perpetrators with female perpetrators, perpetrators recorded significantly higher scores on all six variables. When comparing male non-perpetrators with male perpetrators, perpetrators recorded higher scores on all variables. Score differences were statistically significant, except for alcohol quantity and alcohol-related RMA. When comparing male perpetrators with female perpetrators, results indicated that perpetrators, whether male or female, reported statistically equivalent responses on five variables (alcohol consumption frequency, quantity, alcohol before sex, misperception of sexual intent, and alcohol-related expectancies regarding female sexuality). The only significant difference recorded between male and female perpetrators regarded alcohol-related RMA, for which male perpetrators recorded higher scores than female perpetrators (see Table 2 for complete results).

#### Predictors of Sexual Coercion

For both genders, logistic hierarchical regression analyses were conducted to determine the main effects of alcohol-use and cognitive variables on sexual coercion perpetration. In Step 1, alcohol-use variables (alcohol-use frequency, quantity, and before sex) were entered as predictors. In Step 2, alcohol-use variables were entered along with cognitive factors (misperception of sexual intent, alcohol-related expectancies regarding sexuality, alcohol-related RMA).

For men, Step 1 indicated that alcohol-use before sex was a significant predictor of sexual coercion (*x*^2^(3, 181) = 12.88, *p* < .01). This model explained 7% of the variance in male sexual coercion perpetration (*R*^2^ = .07 (Cox & Snell)). Step 2 indicated that misperception of sexual intent and alcohol-related expectancies regarding female sexuality were significant predictors of sexual coercion in male perpetrators (*x*^2^(4, 181) = 30.70, *p* < .001). More specifically, those who have misinterpreted someone’s actions as indicating sexual intent were 4.35 times more likely to have a history of sexual coercion, and those who expected women to be more sexually receptive under the influence of alcohol were 2.17 times more likely to have a history of sexual coercion. This model explained 21% of the variance in male sexual coercion perpetration, a significantly greater proportion than the first model (*R*^2^ = .21 (Cox & Snell)). Considering this, the second model was retained.

For women, Step 1 indicated that alcohol-use before sex and alcohol-use quantity were significant predictors of female sexual coercion perpetration (*x*^2^(3, 556) = 21.64, *p* < .001). This model explained 4% of the variance in female sexual coercion perpetration (*R*^2^ = .04 (Cox & Snell)). Step 2 indicated that alcohol-use before sex, misperception of sexual intent, and alcohol-related RMA were significant predictors of female sexual coercion (*x*^2^(4, 556) = 20.61, *p* < .001). More specifically, those who drank alcohol at a greater frequency before having sex were 1.45 times more likely to have a history of sexual coercion, those who had misperceived someone’s actions as indicating sexual intent were 2.02 times more likely to have a history of sexual coercion and those who adhered more strongly with alcohol-related rape myths were 1.23 times more likely to have a history of sexual coercion. This model explained 7% of the variance in female sexual coercion perpetration, a significantly greater proportion than the first model (*R*^2^ = .07(Cox & Snell)). Considering this, the second model was retained.

### Discussion

In recent years, significant associations have been established between alcohol-use and sexual coercion. Cognitive factors have been proposed to play an important role in this association. However, heteronormative and gender biases in research have led to a lack of knowledge on female sexual coercion perpetration. This study aimed to investigate gender differences associated with the role of alcohol-use and cognitive factors in sexual coercion perpetration.

#### Group Comparisons

Looking at comparisons between perpetrators and non-perpetrators, for both genders, perpetrators recorded higher mean scores on all study variables. For women, all alcohol-use and cognitive variables allowed significant discrimination between perpetrators and non-perpetrators. For men, perpetrators recorded higher scores on all variables, with all but two variables reaching statistical significance. Given that alcohol-use and cognitive variables were shown to be significant risk factors for male sexual coercion perpetration in previous research, these results partly legitimize the reliability of our sample. For men, we did not record significant between-group differences in terms of alcohol-use quantity. The categorical nature of the variable may have influenced this result, by limiting the range of response options. Similarly, no group differences were recorded for alcohol-related RMA between male perpetrators and non-perpetrators, suggesting that alcohol-related RMA is similar among men, regardless of perpetrator status. The significant difference between male and female perpetrators and male and female non-perpetrators in terms of RMA seems to reflect a general tendency for men to adhere more prominently to attitudes supportive of sexual coercion (Mittal et al., 2017).

It is particularly interesting to note that, besides RMA, there were no significant differences between male perpetrators and female perpetrators on any of the studied variables. Perpetrators, regardless of their gender, seem to exhibit similar alcohol-use, alcohol-related expectancies, and tendencies to misinterpret sexual intent. Since, on the one hand, these factors make it possible to distinguish perpetrators from non-perpetrators, both for women and for men and, on the other hand, these factors are common to all perpetrators, regardless of their gender, they should be considered important targets towards the prevention of sexual coercion.

#### Predictors of Sexual Coercion

For both men and women, a model that considered cognitive variables offered a better fit than a model containing only alcohol-use variables. This supports previous research conducted on male samples which suggested that alcohol-use needs to be considered in combination with cognitive factors to better explain sexual coercion perpetration (Abbey et al., 2004; Benbouriche, 2016). Misperception of sexual intent was a significant predictor for both male and female perpetrators, and was interestingly the only significant predictor that appeared in both models. This suggests that both men and women who have misperceived another’s behavior as indicating sexual intent are more likely to have a history of sexual coercion. For men, our results are consistent with previous research findings (Abbey et al., 1998; Abbey et al., 2001; Farris et al., 2008). To our knowledge, this is the first time, that misperception of sexual intent is investigated in the context of sexual coercion perpetrated by women. In addition to broadening our limited knowledge of female sexual coercion, the fact that misperception of sexual intent is the sole predictor that appears in both models suggests that a person’s inability to perceive, interpret and react to social interactions adequately is a key component towards sexual coercion perpetration. Therefore, underlying elements that affect information processing and behavioral responses in both men and women, such as implicit theories (Ward, 2000) and sexual scripts (Wiederman, 2005) might deserve further investigation. For example, it may be that perpetrators, regardless of gender, adhere more prominently to traditional sexual scripts, which may lead to biases in information processing, perceiving intentions in accordance with their script. This can contribute to more sexual and gender-stereotyped interpretations of social interactions (“men should be able to obtain sex when they want;” “men are always interested in sex and therefore cannot refuse it” “women say no to sex, even when they mean yes”). These explanations are coherent with current explanatory models that emphasize the role of hostile masculinity in male sexual coercion perpetration (Malamuth et al., 1991) and hyperfeminity in female sexual coercion perpetration (Schatzel-Murphy, 2011).

Aside from misperception of sexual intent, only alcohol-related expectancies contributed significantly to the male model of sexual coercion perpetration. Thus, no alcohol-use variable remained significant when cognitive factors were considered. This final model accounted for 21% of the variance in male sexual coercion perpetration. This result seems to indicate that distal measures of alcohol do not explain male sexual coercion beyond their association with other, more relevant risk factors. This highlights the need to investigate cognitive and attitudinal variables in the study of male sexual coercion.

For the female model of sexual coercion perpetration, alcohol-use before sex and alcohol-related RMA (in addition to misperception of sexual intent) were significant predictors of female sexual coercion perpetration. This suggests that women who pair alcohol-use and sex more frequently are more likely to have a history of sexual coercion perpetration. The fact that alcohol-related RMA was a significant predictor for women seems to point again towards the hyperfeminity model suggested by Schatzel-Murphy (2011), and expanded by Parent et al. (2018), which translates into more stereotypical attitudes towards gendered relations, and thus greater RMA. While the regression model did explain a significant proportion of variance in female sexual coercion perpetration, this proportion was considerably smaller in the female model than in the male model (7% compared to 21%). Similarities in risk factors for sexual coercion perpetration in both genders lead us to consider how they might fall within a broader heteronormative framework. At the same time, these results highlight the need to consider other variables towards a better understanding of female sexual coercion perpetration.

#### Implications

On a practical level, the significant prevalence of sexual coercion perpetration in female participants and similarities in risk factors between male and female perpetrators reinforce the importance of including women in prevention and intervention efforts. Common risk factors for sexual coercion perpetration in men and women can be understood from a global, societal point of view. In fact, the present study seems to highlight how this broader context influences attitudes that are linked to sexual coercion in both genders and emphasize the need to avoid the gendered approach that sexual coercion research and prevention has historically taken. In fact, misperception of sexual intent, alcohol expectancies related to female sexuality, and RMA are all anchored in stereotypical attitudes and expectations toward gendered behaviors and relations, which are conveyed in the social heteronormative context in which we evolve. Challenging these heteronormative and gendered biases which contribute to a socio-cultural tolerance for sexual coercion seems to be a particularly promising avenue. Thus, sexual education as well as prevention and intervention efforts for men and women should not only target attitudes specific to sexual coercion, but also more general gendered attitudes and expectations that are anchored in a heteronormative social context. Therapists may explore clients’ sexual scripts and use cognitive restructuring in order to foster more flexible and inclusive views on sexuality and gender roles. On an empirical level, results highlight gaps in our knowledge of female sexual coercion perpetration. While similarities in risk factors towards sexual coercion perpetration in men and women exist, it seems extending male models of sexual coercion to female perpetrators involves some limitations. Results therefore strengthen the necessity to steer away from the male-centered approach that sexual coercion perpetration research has historically taken.

#### Limitations

The results of this study should be considered in light of some limitations. First, because of the cross-sectional and observational design, the present results should be interpreted as indicating associations between variables, but not causality. Experimental and longitudinal studies are needed to study the interaction between alcohol-use, cognitive factors, and sexual coercion in a more dynamic way. Since the data were collected via a self-report questionnaire, which relies on a retrospective account of specific events, it is subject to social desirability and memory biases. This highlights the relevance of conducting experimental studies that can measure such variables in more implicit ways. Finally, although efforts were made to obtain a diversified sample, most participants were students, heterosexual, and Caucasian. Also, in wanting to compare genders, participants who identified neither as men nor women were excluded from the main analyses. Caution should thus be used when generalizing the results to individuals of gender, sexual and ethnic minorities. Results need to be replicated with greater sampling diversity.

### Conclusion

The present study aimed to investigate gender differences associated with the role of alcohol-use and cognitive factors in sexual coercion. Results revealed that alcohol-use (frequency, before sex) and cognitive variables (gendered expectancies towards sexuality, misperception of sexual intent) are interesting prevention and intervention targets, since they allowed to discriminate perpetrators from non-perpetrators in both genders, while being similar among perpetrators of both genders. Misperception of sexual intent seems to be particularly important from a conceptual and practical point of view, as it was the only shared variable in female and male prediction models. As a whole, these results suggest the need to break away from heteronormative and gendered approaches to sexual coercion research, and to not only focus on attitudes specific to sexual coercion in prevention and intervention efforts, but also more general gendered and heteronormative attitudes.

## Notes

1. Throughout the manuscript, the terms male/men and female/women will be used. However, it is important to note that we are referring to participants who identify as men or women, regardless of biological sex.

2. According to Cohen’s power analysis table (1992), sample size allowed the detection of medium effect sizes at a .05 significance level for both ANOVAs and logistic regression analyses.

## Author Biographies

**Véronique Bonneville**, BA, is a PsyD candidate at the University of Quebec in Outaouais and SSHRC grant recipient. Her research interests are centered around identifying risk factors for sexual coercion perpetration.

**Dominique Trottier**, PhD, is a clinical psychologist and professor at the University of Quebec in Outaouais, where she leads the Laboratory for the Study of Delinquency and Sexuality. Her research focuses on risk factors associated with sexual coercion perpetration and sexual recidivism.
